# Evolução Tardia das Próteses Biológicas e Mecânicas em Posição Aórtica

**DOI:** 10.36660/abc.20200135

**Published:** 2021-07-15

**Authors:** Larissa Ventura Ribeiro Bruscky, Carlos Gun, Auristela Isabel de Oliveira Ramos, Alice Lemos Morais

**Affiliations:** 1 Instituto Dante Pazzanese de Cardiologia São PauloSP Brasil Instituto Dante Pazzanese de Cardiologia, São Paulo, SP – Brasil

**Keywords:** Valva Aórtica, Bioprótese/tendências, Implante de Prótese de Valva Cardíaca/complicações, Próteses Valvulares Cardíacas, Febre Reumática

## Abstract

**Fundamento:**

Apesar da constante renovação e do aprimoramento das próteses valvares cardíacas, a decisão sobre substituição por prótese biológica ou mecânica permanece controversa.

**Objetivo:**

Comparar pacientes submetidos à cirurgia para troca valvar aórtica utilizando substituto biológico ou mecânico.

**Métodos:**

Estudo observacional, do tipo coorte histórica por análise de prontuário. Foram selecionados 202 operados entre 2004 e 2008, com seguimento médio de 10 anos. O nível de significância estatística adotado foi de 5%.

**Resultados:**

A média de idade foi de aproximadamente 50 anos para ambos os grupos, com a maioria (70%) do sexo masculino. A probabilidade de sobrevida livre de óbito e reoperação foi significativamente maior nos pacientes com prótese mecânica (HR=0,33; IC 95%=0,13-0,79; p=0,013). Não houve diferença entre os grupos em relação à mortalidade tardia. Por outro lado, o risco de reoperação foi significativamente maior em pacientes tratados com prótese biológica em comparação com a prótese mecânica (HR=0,062; IC 95%=0,008-0,457; p=0,006). O risco de eventos adversos composto de acidente vascular encefálico (AVE), sangramento, endocardite, trombose e regurgitação paraprotética foi semelhante entre os grupos (HR=1,20; IC 95%=0,74-1,93; p=0,44). O risco de sangramento foi significativamente maior em pacientes tratados com prótese mecânica (HR=3,65; IC 95%=1,43-9,29; p=0,0064), porém não houve sangramento fatal.

**Conclusão:**

Não há diferença de mortalidade em 10 anos entre os dois grupos. Há aumento significativo no risco de reoperação ao se optar por próteses biológicas, principalmente para os menores de 30 anos de idade. Já os pacientes portadores de prótese mecânica têm maior risco de sangramento não fatal.

## Introdução

A substituição da valva aórtica é realizada desde a década de 1950.^1^ Desde então, o avanço técnico no projeto da prótese e a otimização do procedimento cirúrgico reduziram o risco de complicações relacionadas ao procedimento, além de melhorar significativamente o prognóstico a longo prazo.^1^

Apesar da constante renovação e aprimoramento das próteses, a decisão sobre substituição por prótese biológica ou mecânica para a posição aórtica permanece controversa. A principal desvantagem da prótese biológica é a degeneração progressiva dos folhetos, porém apresenta uma baixa trombogenicidade com menor tempo de anticoagulação e ausência de ruídos. Já as próteses mecânicas exigem anticoagulação prolongada, modificações substanciais no estilo de vida e carregam um maior risco a longo prazo de tromboembolismo e eventos hemorrágicos.^2^

Existem poucos estudos brasileiros comparativos entre próteses biológicas e mecânicas, bem como poucos são os que descrevem a influência do contexto epidemiológico no desfecho em um período de 10 anos.

No Brasil, a causa mais comum de valvopatia é a febre reumática, logo, os pacientes são submetidos à intervenção cirúrgica em uma faixa etária mais baixa que a população operada em países desenvolvidos.^3^ Além da idade, muitos dos pacientes com valvopatia são originários de regiões pouco favorecidas, portanto têm dificuldade em fazer o controle da anticoagulação.

Dentro desse contexto, o objetivo desse estudo foi avaliar mortalidade, reoperação e eventos adversos em pacientes submetidos à cirurgia para troca valvar aórtica por substituto biológico ou mecânico, em um hospital terciário da rede pública do Estado de São Paulo.

## Métodos

O delineamento do estudo foi observacional, do tipo coorte histórica por análise de prontuário.

### Amostra

Pacientes com idade maior que 18 anos e menor ou igual a 65 anos, submetidos a troca valvar aórtica por prótese biológica ou mecânica, no período de 01 de janeiro de 2004 a 31 de dezembro de 2008, com seguimento médio de 10 anos. Todas as próteses mecânicas utilizadas foram de duplo folheto e todas as biológicas implantadas foram próteses nacionais disponibilizadas pelo Sistema Único de Saúde.

Desfecho primário combinado: tempo de sobrevida livre de reoperação e mortalidade tardia (após 30 dias de cirurgia) por todas as causas. Desfecho secundário: tempo de sobrevida livre de evento adverso, composto de AVC, sangramento, endocardite, trombose e RPP.

Foi avaliado também idade, sexo, etiologia da disfunção da prótese aórtica, além do ritmo cardíaco, uso de anticoagulação e dados ecocardiográficos considerando grau de hipertensão pulmonar, fração de ejeção de ventrículo esquerdo e diâmetros ventriculares.

A escolha da prótese foi realizada a critério do médico cardiologista que acompanhava o paciente, levando em consideração a idade, possibilidade de anticoagulação, características clínicas e contexto socioeconômico.

### Aspectos éticos

Os aspectos clínicos e cirúrgicos durante o período de estudo foram completados a partir de informações escritas nos prontuários dos pacientes, não existindo risco atribuído ao indivíduo. Quanto à privacidade e à confidencialidade, são garantidas a preservação do anonimato dos pacientes e a utilização dos dados obtidos na pesquisa apenas para a finalidade do projeto. O projeto de investigação recebeu aprovação prévia da Comissão de Ética em Pesquisa em Saúde do Instituto Dante Pazzanese de Cardiologia, registrado sob o n° 4864/2018, para obtenção de permissão de execução no âmbito deste hospital.

### Definições

As definições foram obtidas do artigo de Força Tarefa da European Association of Percutaneous Cardiovascular Interventions (EAPCI), European Society of Cardiology (ESC) e European Association for Cardio-Thoracic Surgery (EACTS) e também da Diretriz Brasileira de Valvopatia de 2011 com atualização de 2017.

### Análise estatística

As variáveis quantitativas foram descritas por média e desvio padrão, e as qualitativas por meio de frequências absolutas e relativas.

A comparação entre os grupos na fase de balanceamento foi realizada pelo teste t-Student para amostras independentes, em caso de variáveis quantitativas, e teste exato de Fisher, para as variáveis qualitativas (taxas e proporções).

A análise descritiva foi realizada no Excel 2004 e o teste de normalidade (Shapiro Wilk) pelo SPSS com nível de significância de 5%.

Para avaliar o tempo de sobrevida, o tempo de sobrevida livre de reoperação e tempo de sobrevida livre de eventos adversos (AVC, sangramento, endocardite, trombose e RPP) foi utilizada a curva de Kaplan-Meier. Foi aplicado, para comparar as curvas entre os grupos, o teste de Log-rank. Para analisar os desfechos foi utilizado o modelo de riscos proporcionais de Cox. Não foi considerada a análise múltipla de variáveis pois o modelo de seleção *stepwise* resultou no próprio modelo de riscos proporcionais simples de Cox. Como medida de efeito, foi calculada a razão entre taxas instantâneas de incidências ( *hazard ratio* ), com seus respectivos intervalos, com 95% de confiança. O nível de significância estatística adotado foi de 5%. Nos cálculos atuariais, empregou-se o programa R Core Team 2019.

Foi utilizado o teste exato de Fisher para avaliar diferenças na taxa de mortalidade precoce entre os grupos, considerando a soma dos pacientes elegíveis e os seis excluídos por óbito em menos de 30 dias do procedimento. Esses seis pacientes não foram considerados nas demais análises. A análise está robusta aos dados censurados, pois o valor de p continua não significativo mesmo considerando todos os excluídos por falta de seguimento maior de 30 dias, como óbito precoce ou não óbito precoce.

Para análise da taxa de reoperação nos subgrupos divididos por idade (18-29 anos / 30-49 anos / ≥ 50 anos) foi utilizado o teste de Bonferroni com referência para o valor de p ajustado para 0,05/3 = 0,016666.

## Resultados

### Amostra

**Figura 1:**
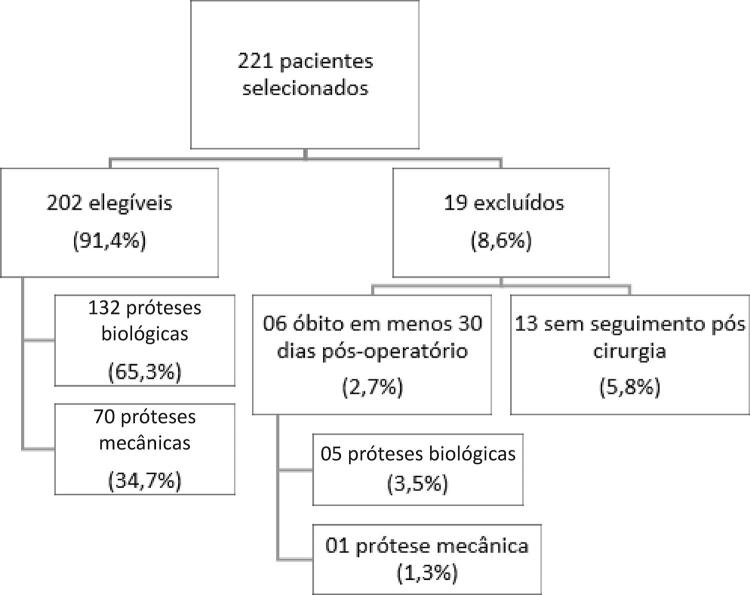
– Distribuição dos pacientes.

Foram avaliados os dados de 221 pacientes submetidos à troca da valva aórtica isolada. A mortalidade hospitalar, considerada até 30 dias de pós-operatório foi de 2,7%, 6 pacientes do total de 221 da amostra. Treze pacientes tiveram perda de seguimento, correspondente a 5,8% da amostra total. Foram então contabilizados 202 pacientes elegíveis, dos quais 132 pertencentes ao grupo de biopróteses (65,3%), sendo 126 (95,5%) prótese porcina e 6 (4,5%) prótese de pericárdio bovino, e 70 ao grupo de prótese mecânica (34,7%). Dados demonstrados na figura 1 .

A média de seguimento foi de 9,29 ± 3,8 anos, sendo que 74% dos pacientes foram acompanhados por mais de oito anos. O tempo de máximo evolução foi 14,25 anos para próteses biológicas e 14,34 anos para prótese mecânica.

As características basais dos pacientes com bioprótese e prótese mecânica foram semelhantes e podem ser observadas na tabela 1 . O uso de anticoagulação, como esperado, foi mais prevalente no grupo submetido à troca da valva aórtica por prótese mecânica, com p < 0,001. Todos os outros fatores não foram estatisticamente diferentes entre os dois grupos: idade, sexo, etiologia da disfunção valvar, ritmo cardíaco e dados do ECO pré-operatório (DDVE, DSVE, FEVE, PSAP).

**Tabela 1:** – Caracterização da amostra

Variável	Grupo estudado		Valor de p*
	Prótese biológica	Prótese mecânica	
	(N= 132)	(N= 70)	
**Idade ± DP**	50,78±11,67	47,67±14,09	0,116
**Sexo - n (%)**			0,504
Masculino	75,8%	71,4%	
Feminino	24,2%	28,6%	
**Etiologia - n (%)**			0,357
Degenerativa	62,1%	57,1%	
Reumática	21,9%	22,9%	
Dilatação Aorta	12,1%	10%	
Bicúspide	3,9%	10%	
**Ritmo ECG - n (%)**			0,568
Sinusal	80,3%	84,3%	
FA/Flutter	19,7%	15,7%	
**Uso de ACO - n (%)**			< 0,001
Sim	12,1%	97,1%	
Não	87,9%	2,9%	
**ECO pré-operatório**			
PSAP ± DP			0,551
Sem HP	78%	82,6%	
HP leve	16,7%	11,6%	
HP moderada	3,8%	5,8%	
HP grave	1,5%	0%	
FEVE ± DP	58,51±12,71	61,68±10,9	0,067
DSVE ± DP	41,01±11,79	40,06±11,28	0,547
DDVE ± DP	60,92±11,68	60,74±12,7	0,923

* Valor de p<0,05 considerado estatisticamente significativo. DP: desvio padrão; ECG: eletrocardiograma; FA: fibrilação atrial; ACO: anticoagulação com Marevan; ECO: ecocardiograma; PSAP: pressão sistólica de artéria pulmonar; HP: hipertensão pulmonar; FEVE: fração de ejeção de ventrículo esquerdo; DSVE: diâmetro sistólico do ventrículo esquerdo; DDVE: diâmetro diastólico do ventrículo esquerdo.

A mortalidade precoce (com menos de trinta dias pós cirurgia) foi de 2,7% que corresponde a seis pacientes de um total de 221 analisados. Não houve diferença na mortalidade precoce entre os dois tipos de substituto (1,3% prótese mecânica *versus* 3,5% prótese biológica; p = 0,666). Como o objetivo deste estudo foi comparar os pacientes com os dois tipos de prótese (biológica e mecânica) a longo prazo, esses seis pacientes foram excluídos da análise.

### Dados de sobrevida e reoperação

**Figura 2:**
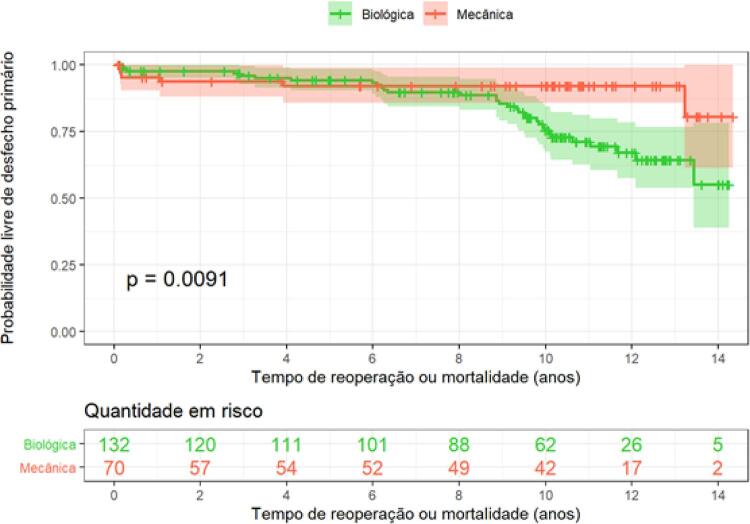
– Curva de Kaplan-Meier para avaliar probabilidade de sobrevida livre de eventos (óbito ou reoperação).

A probabilidade de sobrevida livre de óbito por qualquer causa e reoperação foi significativamente maior em pacientes tratados com prótese biológica em comparação com a prótese mecânica (HR= 0,33; 95% intervalo de confiança [IC] 0,13-0,79; p= 0,013), como observado na figura 2 .

Foram observados oito óbitos num período de 10 anos no grupo de pacientes portadores de prótese biológica e cinco no grupo de prótese mecânica. Esses dados correspondem a um percentual ajustado de 6,11% de mortalidade para o grupo de prótese biológica e 7,9% para prótese mecânica (p=0,68).

Por outro lado, a análise de reoperação isolada evidenciou uma diferença significativa a favor da prótese mecânica (HR=0,062; IC= 0,008-0,457; p=0,006). Num período de 10 anos, dezenove pacientes portadores de prótese biológica foram reoperados, correspondendo a um percentual de 21,24%. Não teve nenhum evento registrado para o grupo de prótese mecânica.

**Figura 3:**
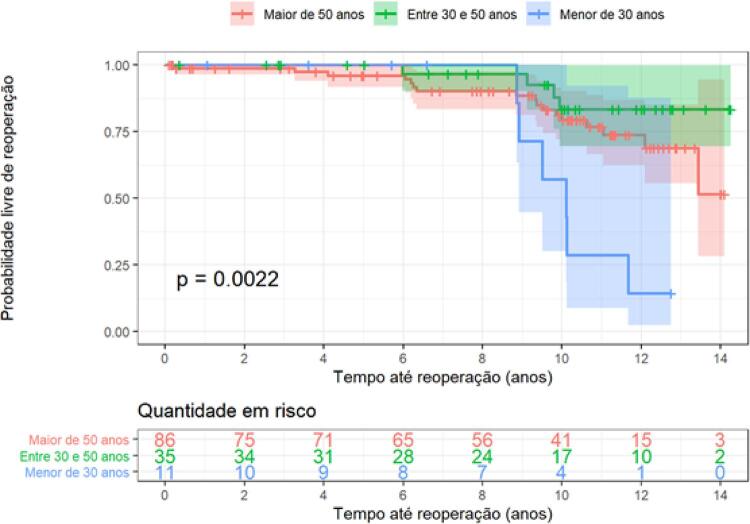
– Curva de Kaplan-Meier para avaliar probabilidade de sobrevida livre de reoperação separados por subgrupos de >18 a <30 anos, ≥ 30 a 49 anos e ≥ 50 anos.

A reoperação foi avaliada de acordo com a faixa etária, separados por subgrupos <30 anos, entre 30 e 49 anos e ≥50 anos. A probabilidade livre de reoperação foi menor para o grupo <30 anos em comparação com o grupo entre 30-49 anos (HR= 6,69; IC= 1,88-23,8; p=0,003) e também em comparação com o grupo ≥ 50 anos (HR= 3,51; IC= 1,37-9,03; p=0,008). Por sua vez, o grupo ≥50 anos não teve diferença em relação ao grupo entre 30-49 anos (HR= 0,50; IC= 0,16-1,50; p=0,219). Esses dados podem ser observados na figura 3 .

### Dados de eventos adversos

**Figura 4:**
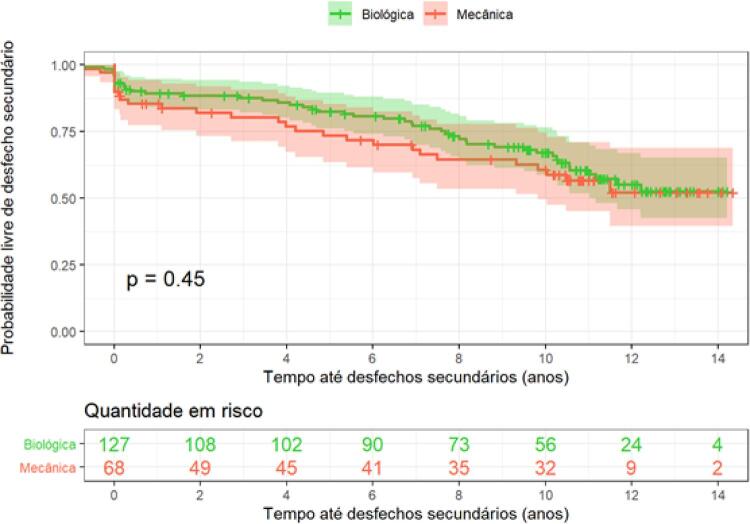
– Curva de Kaplan-Meier para avaliar probabilidade de sobrevida livre de desfechos secundários (AVC, sangramento, endocardite, trombose e RPP).

O desfecho secundário composto de AVC, sangramento, endocardite, trombose e RPP foi semelhante nos dois grupos (HR=1,20; IC 95%= 0,74-1,93; p=0,44), como observado na figura 4 .

**Figura 5:**
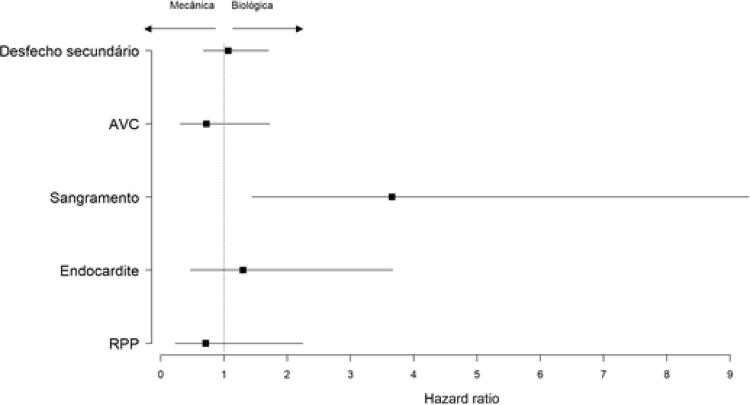
– Gráfico de Forest Plot do efeito dos eventos adversos em relação ao tipo de prótese (mecânica ou biológica).

O gráfico de Forest plot abaixo ( figura 5 ) representa a análise do desfecho secundário realizado pelo *hazard ratio.*

O risco de sangramento foi significativamente maior em pacientes tratados com prótese mecânica em comparação com a prótese biológica (HR=3,65; IC 95%= 1,43-9,29; p=0,0064). Foi observado um percentual ajustado pelos dados censurados de sangramento em 10 anos de 5,38% de sangramento para o grupo de prótese biológica e 20,97% para o grupo de prótese mecânica.

A taxa de AVC em 10 anos foi de 14.10% para o grupo de prótese biológica e 11.56% para o grupo de prótese mecânica, p=0,47.

O risco de RPP foi semelhante entre o grupo de prótese biológica e prótese mecânica (HR=0,71; IC 95%= 0,22-2,24; p=0,56). Foi observado um percentual ajustado pelos dados censurados em 10 anos de 6.53% de RPP para o grupo de prótese biológica e 3.38% para o grupo de prótese mecânica.

O risco de endocardite foi semelhante entre o grupo de prótese biológica e prótese mecânica (HR=1,30; IC 95%= 0,46-3,66; p=0,61). Foi observado um percentual ajustado pelos dados censurados em 10 anos de 6.12% de endocardite para o grupo de prótese biológica e 1.57% para o grupo de prótese mecânica.

O risco de trombose foi semelhante entre o grupo de prótese biológica e prótese mecânica (p=0,1). Foi observado um percentual ajustado pelos dados censurados em 10 anos de 5.06% de trombose para o grupo de prótese biológica não teve nenhum evento registrado para o grupo de prótese mecânica.

A taxa de DPP observada foi de 3,78% para o grupo de prótese biológica e não teve nenhum evento registrado para o grupo de prótese mecânica. A ausência de evento no grupo da prótese mecânica não permitiu uma análise estatística mais detalhada. Dados demostrados na Tabela 2.

## Discussão

Mais de 30 anos após a introdução das próteses modernas, a escolha entre biológicas e mecânicas permanece controversa. São poucos os trabalhos randomizados, controlados e com grande número de pacientes para guiar de forma definitiva a escolha da melhor prótese. O nível de evidência na maioria das recomendações das diretrizes é baixo (nível C), ficando a escolha baseada em dados limitados, na experiência clínica e no bom senso. Espera-se que este trabalho auxilie no conhecimento acerca da evolução dos tipos de prótese para essa amostra específica que nos deparamos.

No presente estudo buscamos avaliar a cirurgia de troca da valva aórtica por prótese biológica ou mecânica em um grupo de pacientes atendidos pelo Sistema Único de Saúde (SUS). A doença degenerativa foi a principal causa de valvopatia, seguida pela doença reumática com cerca de 22% dos casos. Esse predomínio epidemiológico da doença degenerativa é semelhante a países desenvolvidos, porém o motivo da etiologia reumática não ter sido mais expressiva pode ser justificado pela seleção de pacientes com limite de idade superior a 18 anos e de se tratar de um estudo exclusivo da valva aórtica.^4^

Chama atenção que cerca de 80% dos pacientes, nos dois grupos, apresentavam-se em ritmo sinusal. Cerca de 19,7% dos pacientes do grupo de prótese biológica apresentavam fibrilação atrial, porém apenas 12,1% estavam em uso de anticoagulação. Dois pacientes do grupo de prótese mecânica também estavam sem uso de anticoagulação, apesar das orientações e risco de trombose, sendo que um evoluiu para óbito e o outro para AVC. Esses dados refletem a dificuldade de realização de anticoagulação na população menos favorecida do país. Não houve diferença estatisticamente significativa dos parâmetros ecocardiográficos pré-operatórios. A maioria dos pacientes apresentava função ventricular preservada e sem hipertensão pulmonar importante.

A coorte estudada constitui uma amostra com média de idade de 50 anos. O risco de reoperação foi significativamente maior em pacientes com bioprótese, principalmente para os pacientes menores de 30 anos. As curvas começam a se distanciar a partir do quarto ano do implante da prótese e tornam-se mais evidentes após os 8 anos, sendo que 50% dos pacientes com menos de 30 anos já tinham a indicação de reoperação em 10 anos de evolução. Em uma média de 10 anos houve uma única indicação de reoperação no grupo de prótese mecânica. Da mesma forma Hammermeister et al. encontraram maior número de intervenções no grupo que recebeu prótese valvar biológica em posição aórtica em comparação com os que receberam prótese mecânica (29% *versus* 10%; p = 0,004).^5^ Vale ressaltar que as taxas de reoperação não capturam totalmente o risco de degeneração estrutural da prótese, pois alguns pacientes com deterioração estrutural significativa não são considerados candidatos à reoperação devido ao alto risco cirúrgico.

A mortalidade tardia foi semelhante entre os dois grupos, com valores de percentual ajustado muito próximos, 6,11% para prótese biológica e 7,93% para prótese mecânica, p = 0,68. Estudos mais recentes produzem resultados mistos, com uma tendência a menores taxas de mortalidade com prótese mecânica em grupos etários mais jovens (menores que 55 anos).^6^

Sangramento ocorreu nos dois grupos, visto que 97,1% dos portadores de prótese mecânica estavam em uso de anticoagulação e 12,1% do grupo de prótese biológica, embora nos portadores de prótese mecânica tenha sido significativamente maior (p = 0,0064). Não foi registrado nenhum sangramento fatal ou AVC hemorrágico. Como exemplo, no Veterans Affairs Cooperative Study, 575 pacientes foram aleatoriamente designados para terapia com uma prótese mecânica ou biológica. A probabilidade de sangramento em 11 anos foi significativamente maior nas válvulas mecânicas (42% *versus* 26%).^7^

Na presente coorte, não houve diferença estatisticamente significativa no risco de endocardite. Durante o primeiro ano de pós-operatório, a infecção geralmente ocorre com igual frequência nos dispositivos mecânicos e biológicos. Durante o seguimento a longo prazo, as taxas de endocardite com próteses biológicas são semelhantes ou ligeiramente superiores às das próteses mecânicas, porém os dados disponíveis ainda são muito limitados.^8^

A literatura demonstra maior taxa de trombose de prótese em pacientes com prótese mecânica *versus* prótese biológica e destaca a necessidade de ser manter terapia anticoagulante contínua nesses pacientes.^9^ Esse dado vai de encontro ao observado no presente estudo, em que não houve diferença estatisticamente significativa entre as duas próteses com valores de percentual ajustado de 5,06% para prótese biológica e nenhum caso registrado para prótese mecânica. Esse dado pode ser justificado pelo atendimento dos pacientes do estudo em centro especializado de anticoagulação. Para o controle de anticoagulação, Chiquette et al.,^10^ comparam o atendimento convencional em consultório com o acompanhamento em clínicas especializadas. Nas clínicas especializadas foram encontradas menores taxas de ocorrência de eventos tromboembólicos (maiores, menores e fatais).^10^

Não houve diferença de risco de AVC isquêmico entre os dois grupos estudados (percentual ajustado de 14,1% para a prótese biológica *versus* 11,5% para prótese mecânica aos 10 anos; p = 0,47). Na literatura encontramos dados em que o risco de complicações tromboembólicas é geralmente semelhante ou menor em pacientes com prótese biológica em comparação com pacientes com prótese mecânica tratada com anticoagulação. Em um exemplo de um estudo observacional, o risco cumulativo de AVC entre pacientes de 45 a 54 anos de idade submetidos a troca da valva aórtica foi significativamente menor entre aqueles com prótese biológica em comparação com prótese mecânica (aproximadamente 10% *versus* 16% aos 15 anos; HR = 0,64; IC 95% 0,46-0,86; p < 0,05).^6^ A elevada taxa de AVC encontrada nessa coorte possivelmente se deve ao fato de a maioria dos pacientes ter alta prevalência de comorbidades associadas, interferindo nas taxas de AVC em ambos os grupos e também pela ausência de anticoagulação em pacientes em ritmo de fibrilação atrial e ausência de dados de controle do tempo de protrombina dos pacientes analisados.

Não houve diferença estatisticamente significativa para o risco de RPP entre as duas próteses em posição aórtica. Foi observado um percentual ajustado pelos dados censurados em 10 anos de 6,53% de RPP para o grupo de prótese biológica e 3,38% para o grupo de prótese mecânica, dados corroborados pela literatura que estima uma incidência de 2% a 10% na posição aórtica. Como exemplo, em estudos com ecocardiografia transesofágica (ETE) após cirurgias de troca valvar, a incidência de RPP variou de 3% a 6% com uma tendência estatística a maior prevalência em próteses mecânicas.^11^

A DPP foi observada em 3,78% dos indivíduos com prótese biológica e em nenhum paciente do grupo de prótese mecânica, possibilitando apenas uma análise simples descritiva. Esse número ficou aquém do evidenciado na literatura, em que pode chegar a cerca de 20% a 70%.^12^De acordo com a revisão sistemática da European Heart Journal acerca do impacto a longo prazo do DPP, que avaliou 34 estudos com um total de 27186 pacientes, sua presença esteve associada a um decréscimo de sobrevida a longo prazo (HR = 1,34, 95% CI = 1,18-1,51).^13^ Quando comparada a incidência entre prótese mecânicas e biológicas, a DPP parece ter maior probabilidade de ocorrer com biopróteses, já que próteses mecânicas geralmente têm maiores orifícios valvares efetivos comparados com próteses biológicas devido ao espaço ocupado pelas suas hastes de suporte. Em pacientes com anel aórtico pequeno, o tamanho do orifício valvar efetivo é crucial para otimizar o desempenho hemodinâmico da prótese e, assim, evitar a ocorrência de DPP. Em alguns casos, pacientes com um pequeno anel podem se beneficiar de uma prótese mecânica.^1^

No presente estudo foi encontrada uma média de 202 dias entre a indicação da cirurgia e sua realização, porém com um padrão de distribuição bastante heterogêneo. Os diferentes perfis de indicações cirúrgicas e características dos pacientes justificam essa heterogeneidade e constituem uma contribuição importante no tempo de espera do paciente pela cirurgia.

### Limitações do estudo

Este trabalho é um estudo não randomizado. Seus dados têm validação externa restrita, porém seus resultados podem servir de base para outros estudos analíticos e prospectivos, a fim de se obter dados mais consistentes. Também tem como limitação sua realização em um único centro, amostra insuficiente para eventos raros e perda de seguimento.

Neste estudo não foi avaliada a mortalidade relacionada à reoperação, o que talvez subestime a mortalidade do grupo de bioprótese. Também não foi avaliado o controle do tempo de protrombina dos pacientes em uso de anticoagulação, o que dificulta compreender melhor a elevada incidência de AVC isquêmico nos dois grupos.

## Conclusão

A probabilidade de sobrevida livre de óbito e reoperação, em pacientes com média de idade de 50 anos, operados em hospital terciário do SUS do Estado de São Paulo, foi significativamente maior nos pacientes com prótese mecânica, às custas da maior durabilidade da prótese. Não houve diferença de mortalidade em 10 anos entre os dois grupos. A necessidade de reoperação foi significativamente maior nos pacientes com bioprótese e com idade inferior a 30 anos. Não houve diferença entre os grupos em relação aos eventos adversos combinados. Embora não tenha havido nenhum sangramento fatal, pacientes com prótese mecânica apresentaram mais sangramento.
