# Supplementary material for: Evolução Tardia das Próteses Biológicas e Mecânicas em Posição Aórtica
Source: Arq Bras Cardiol. 2021 Jul 15;117(1):28–36. [Article in Portuguese] doi: 10.36660/abc.20200135 (PMC8294736; doi:10.36660/abc.20200135)
Supplement: Translation

# Translation: Late Outcomes of Aortic Valve Replacement with Bioprosthesis and Mechanical Prosthesis

**Authors:** Larissa Ventura Ribeiro Bruscky, Carlos Gun, Auristela Isabel de Oliveira Ramos, Alice Lemos Morais

**Affiliations:** 1 Brasil Instituto Dante Pazzanese de Cardiologia, São Paulo, SP – Brasil

**Keywords:** Aortic Valve, Bioprosthesis/trends, Heart Valve Prosthesis Implantation/complications, Heart Valve Prosthesis, Rheumatic Fever

## Abstract

**Background:**

Despite constant improvement and refinement of the prostheses, the decision between mechanical and biological valves for aortic valve replacement is still controversial.

**Objective:**

To compare outcomes of aortic valve replacement with bioprosthesis and mechanical prosthesis.

**Methods:**

This was an observational, historical cohort study with review of medical records. A total of 202 patients who underwent heart valve replacement surgery between 2004 and 2008 were selected, with a mean follow-up of 10 years. The level of significance set at 5%.

**Results:**

Mean age of patients was approximately 50 years; most patients were male (70%). Overall mortality- and reoperation-free survival was significantly higher in patients with mechanical prosthesis (HR=0.33; 95%CI=0.13-0.79; p=0.013). No difference was found in late mortality between the two groups. On the other hand, the risk of reoperation was significantly higher in patients with bioprosthesis than mechanical prosthesis (HR=0.062; 95%CI=0.008-0.457; p=0.006). The risk of composite adverse events – stroke, bleeding, endocarditis, thrombosis and paravalvular leak – was similar between the groups (HR=1.20; 95%CI= 0.74-1.93; p=0.44). The risk of bleeding was significantly higher in patients with mechanical prosthesis (HR=3.65; 95%CI= 1.43-9.29; p = 0.0064), although no case of fatal bleeding was reported.

**Conclusion:**

No difference in 10-year mortality was found between the groups. The risk of reoperation significantly increases with the use of bioprosthesis, especially for patients younger than 30 years. Patients with mechanical prosthesis are at increased risk of nonfatal bleeding.

## Introduction

Surgical aortic-valve replacement has been performed since the 1950s.^1^Since then, technical advances in prosthetic manufacturing and optimization in surgical procedure have reduced the risk of complications related to the procedure, and significantly improved long-term prognosis.^1^

Despite constant improvement and refinement of the prostheses, the decision between mechanical and biological valves for aortic valve replacement is still controversial. The main disadvantage of biological prostheses is deterioration of the leaflets; in contrast, compared with mechanical prostheses, bioprostheses are less thrombogenic, requiring lower time of anticoagulation, and do not produce any sounds. On the other hand, mechanical prostheses require long anticoagulation therapy, significant lifestyle changes and impose a higher risk of thromboembolic and hemorrhagic events in long term.^2^

There are few studies in Brazil comparing the performance of biological and mechanical prostheses and describing the influence of epidemiological parameters on 10-year outcomes.

In Brazil, rheumatic fever is the main cause of valvular heart disease and, compared with developed countries, patients undergo surgical intervention at a younger age.^3^In addition, many patients with valvular heart disease come from low-income backgrounds and hence likely to have a poor anticoagulation control.

The aim of the present study was to assess mortality, reoperation, and adverse events in patients undergoing aortic valve replacement surgery with mechanical or biologic valve prosthesis in a São Paulo State public tertiary hospital.

## Methods

This was an observational, historical cohort study with review of medical records.

### Study sample

The study sample was composed of patients aged between 18 and 65 years, who underwent an aortic valve replacement surgery with mechanical or biologic valve prosthesis between January 01, 2004 and December 31, 2008, with a mean follow-up of 10 years. All mechanical prostheses were two-leaflet prosthetic heart valves, and all bioprostheses were national prostheses available in the Brazilian national unified health system.

The combined primary outcome was reoperation-free survival and late all-cause mortality (30 days after surgery). Secondary outcome: event-free survival time, composed of stroke, bleeding, endocarditis, thrombosis and paravalvular leak.

In addition, age, sex, aortic valve dysfunction, heart rhythm, use of anticoagulation, and echocardiographic data (degree of pulmonary hypertension, left ventricular ejection fraction, and ventricular diameters) were also evaluated. The choice of prosthesis was left to the discretion of the treating cardiologist, considering patient’s age, clinical features, socioeconomic status, and possible anticoagulation.

### Ethical aspects

Clinical and surgical aspects during the study period were collected from patients’ medical records. Regarding privacy and confidentiality, participants’ anonymity was ensured, and information gathered during the study was used only for the study purposes. The study was approved by ethics committee of Dante Pazzanese Institute of Cardiology (registration number 4864/2018).

### Definitions

Definitions used in this study followed the European Association of Percutaneous Cardiovascular Interventions (EAPCI), the European Society of Cardiology (ESC), and the European Association for Cardio-Thoracic Surgery (EACTS) guidelines, and the 2017 Update of the Brazilian Guidelines on Valvular Heart Disease.

### Statistical analysis

Quantitative variables were described as mean and standard deviation, and qualitative variables as absolute and relative frequencies.

For group comparisons, the Student’s t-test for independent samples was used for quantitative variables, and the Fisher’s exact test for qualitative variables (rates and proportions). The Kaplan-Meier curve was used for analysis of survival time, reoperation-free survival time, and event-free survival time (stroke, bleeding, endocarditis, thrombosis and paravalvular leak), and the Log-rank test used for comparisons of the curves between the groups.

Analysis of the outcomes was made using the cox proportional hazards model. A multiple regression analysis of variables was not performed, since the stepwise selection model resulted in a simple Cox proportional hazards regression model itself. For measurement of effect, the instantaneous incidence rate (hazard ratio) and respective 95% confidence interval (95%CI) was calculated. Significance level was set at 5%. Data were analyzed with the support of the statistical programming environment R (R Core Team, 2019).

Differences in early mortality between the groups were compared using Fisher’s exact test, considering the number of eligible patients and six patients were excluded for death within 30 days of surgical procedure. These six patients were not considered in the analysis. The analysis was robust to this censoring assumption, since the p-value remained non-significant, even when all patients excluded for loss to follow-up (absent for more than 30 days), were considered as ‘early death’ or ‘no early death’.

For analysis of reoperation rate across age subgroups (18-29 years / 30-49 years/ ≥ 50 years), the Bonferroni test was used, with an adjusted p-value of 0.05/3 = 0.016666.

## Results

### Study sample

A total of 221 patients who underwent aortic valve replacement alone were studied. Thirty-day postoperative mortality was 2.7% (n=6). Thirteen patients (5.8%) were lost to follow-up. Then, 202 patients were considered eligible; 132 (65.3%) with bioprosthesis – 126 of them (95.5%) with a porcine bioprosthesis and six (4.5%) with a bovine pericardium bioprosthesis – and 70 (34.7%) with a mechanical prosthesis as described in Figure 1 .

**Figure 1:** – Flowchart of patient selection

Mean follow-up was 9.3 ± 3.8 years, median of 10.45 years; 74% of patients were followed for more than eight years. The maximum duration of follow-up was 14.25 years for bioprostheses and 14.34 years for mechanical prostheses.

Baseline characteristics were similar between patients with bioprosthesis and mechanical valve prosthesis ( Table 1 ). As expected, the use of anticoagulation was more prevalent in the group of patients who underwent aortic valve replacement with a mechanical prosthesis (p<0.001). No difference between the groups was found in any other variables - age, sex, cause of valve dysfunction, heart rhythm or echocardiographic parameters (PASP: pulmonary artery systolic pressure; PH: pulmonary hypertension; LFEF: left ventricular ejection fraction; LVESD: left ventricular end-systolic diameter; LVEDD: LV end-diastolic diameter).

**Table 1:** – Characteristics of the study groups

Variable	Study groups		p-value*
	Bioprosthesis	Mechanical prosthesis	
	(N= 132)	(N= 70)	
**Age ± SD**	50.78±11.67	47.67±14.09	0.116
**Sex - n (%)**			0.504
Male	75.8%	71.4%	
Female	24.2%	28.6%	
**Etiology - n (%)**			0.357
Degenerative	62.1%	57.1%	
Rheumatic	21.9%	22.9%	
Aortic dilatation	12.1%	10%	
Bicuspid	3.9%	10%	
**ECG rhythm- n (%)**			0.568
Sinus	80.3%	84.3%	
AF/Atrial flutter	19.7%	15.7%	
**Anticoagulant therapy – (Marevan) n (%)**			< 0.001
Yes	12.1%	97.1%	
No	87.9%	2.9%	
**Preoperative ECHO**			
PASP ± SD			0.551
Without PH	78%	82.6%	
Mild PH	16.7%	11.6%	
Moderate PH	3.8%	5.8%	
Severe PH	1.5%	0%	
LVEF ± SD	58.51±12.71	61.68±10.9	0.067
LVESD ± SD	41.01±11.79	40.06±11.28	0.547
LVEDD ± SD	60.92±11.68	60.74±12.7	0.923

* p<0.05 SD: standard deviation; ECG: electrocardiogram; AF: atrial fibrillation; PASP: pulmonary artery systolic pressure; PH: pulmonary hypertension; LFEF: left ventricular ejection fraction; LVESD: left ventricular end-systolic diameter; LVEDD: left ventricular end-diastolic diameter; ECHO: echocardiogram.

No difference in early mortality rate was seen between the two types of valve prostheses (1.3% mechanical prosthesis versus 3.5% biological prosthesis; p = 0.666). Six of 221 (2.7%) patients died less than 30 days after surgery and were excluded from the analysis since the aim of this study was to compare the performance of the prostheses in long term.

### Survival and reoperation data

All-cause survival rate was significantly higher in patients with bioprosthesis compared with those with mechanical prosthesis (HR= 0.33; 95%CI 0.13-0.79; p= 0.013) ( Figure 2 ).

**Figure 2:** – Kaplan-Meier curve for primary outcome-free survival (death or reoperation).

In a ten-year period, eight patients with bioprosthesis and five patients with mechanical prosthesis died, corresponding to an adjusted percentage of 6.1% and 7.9%, respectively, respectively (p=0.68).

On the other hand, the analysis of reoperation alone revealed a significant difference in favor of mechanical prosthesis (HR=0.062; 95%CI = 0.008-0.457; p=0.006). In ten years, 19 (21.24%) patients with bioprosthesis were reoperated, whereas no event was recorded in the mechanical prosthesis group.

Reoperation was analyzed according to subgroups of age – <30 years, between 30 and 49 years and ≥50 years. Probability of reoperation was higher in patients younger than 30 years compared with those aged 30-49 years (HR= 6.69; 95%CI=1.88-23.8; p=0.003) and patients aged ≥ 50 years (HR= 3.51; CI95% = 1.37-9.03; p=0.008). No difference was observed between patients ≥50 years and those aged 30-49 years (HR= 0.50; 95%CI= 0.16-1.50; p=0.219) ( Figure 3 ).

**Figure 3:** – Kaplan-Meier curve for reoperation-free survival by age groups (18-29 years, 30 - 49 years and ≥ 50 years.

### Adverse events

Secondary outcome composed of stroke, bleeding, endocarditis, thrombosis and paravalvular leak was similar between the two groups (HR=1.20; 95%CI= 0.74-1.93; p=0.44), as illustrated in Figure 4 .

**Figure 4:** – Kaplan-Meier curve for secondary outcome-free survival (stroke, bleeding, endocarditis, thrombosis and paravalvular leak).

Results of the analysis of secondary outcome by the hazard ratio was represented by a forest plot ( Figure 5 ).

**Figure 5:** – Forest Plot of adverse effects by type of heart valve prosthesis (mechanical or biological).

The risk of bleeding was significantly higher in patients with mechanical prosthesis than patients with bioprosthesis (HR=3.65; 95%CI = 1.43-9.29; p=0.0064). After adjustment for censored data, the 10-year risk of bleeding was 5.38% in patients with bioprosthesis, and 20.97% in patients with mechanical prosthesis.

The rate of stroke in 10 years was 14.10% for the group of biological prosthesis and 11.56% for the group of mechanical prosthesis (p=0.47). The risk of paravalvular leak was similar between patients with biological and mechanical heart valve prostheses (HR=0.71; 95%CI= 0.22-2.24; p=0.56). The 10-year rate of paravalvular leak, adjusted for censored data, was 6.53% for patients with bioprosthesis and 3.38% for patients with mechanical valve.

The risk of endocarditis was similar between the groups (HR=1.30; 95%CI= 0.46-3.66; p=0.61). The 10-year rate of endocarditis, adjusted for censored data, was 6.12% for patients with bioprosthesis and 1.57% for patients with mechanical valve prosthesis.

The risk of thrombosis was similar between the groups (=0.1). The 10-year rate of thrombosis, adjusted for censored data, was 5.06% for patients with bioprosthesis and no event was recorded in the group with mechanical valve prosthesis.

The observed rate of paravalvular leak identified in the first echocardiography was 3.78% (n=5) for patients with biological valve and no event was recorded in the group with mechanical valve. A more detailed statistical analysis was not possible since no event was recorded in the group of mechanical valve prosthesis.

## Discussion

More than 30 years have passed since the introduction of modern heart valve prostheses, and the choice between mechanical and biological valves remains controversial. There are few randomized, controlled studies, involving a large number of patients, to guide the selection of the best prosthesis. The level of evidence in most guidelines is low (level C), and the selection of prosthesis has depended on limited data, clinical experience, and common sense. We hope that this study will add knowledge about the performance of mechanical and biological heart valve prostheses in this specific group of patients.

In the present study, we aimed to assess the outcomes of aortic valve replacement with a biological or a mechanical prosthesis in a group of patients who use public healthcare services in Brazil. Degenerative disease was the main cause of valvulopathy, followed by rheumatic disease, which accounted for nearly 22% of the cases. This predominance of degenerative disease is similar to that in developed countries; however, the relatively modest percentage of rheumatic disease may be explained by the fact that we included patients aged at least 18 years and that this is a study on isolated aortic valve disease.^4^

It is of note that approximately 80% of total patients were in sinus rhythm; although 19.7% of patients with bioprosthesis had atrial fibrillation, only 12.1% were receiving anticoagulation treatment. Despite recommendations and the risk of thrombosis, two patients with mechanical valve prosthesis were using anticoagulants; one had stroke and the other died. These findings reflect the difficulty of performing anticoagulation in less privileged groups. No statistically significant difference was found in the preoperative echocardiographic parameters between patients with biological and mechanical valve prostheses. Most patients had preserved left ventricular function and did not have severe pulmonary hypertension.

Mean age of patients was 50 years. The risk of reoperation was significantly higher in patients with bioprosthesis, mainly in those younger than 30 years. The distance between the curves becomes larger in the fourth year of valve prosthesis implantation, more evidently after eight years of surgery, when 50% of patients younger than 30 years already had indication for reoperation. In a mean of 10 years, only one patient with mechanical prosthesis required reoperation. In accordance with these findings, Hammermeister et al.^5^reported a greater number of interventions in patients with a bioprosthesis compared with a mechanical prosthesis for aortic valve replacement (29% *versus* 10%; p = 0.004).^5^It is worth mentioning that reoperation rates do not accurately reflect the likelihood of structural valve degeneration, since some patients with significant structural deterioration are not candidates for reoperation due to high surgical risk.

Late mortality was similar between the two groups, with similar adjusted rates – 6.11% in patients with bioprosthesis and 7.93% in patients with mechanical prosthesis (p=0.68). However, more recent studies have reported mixed results, with a trend of lower mortality rates in patients with mechanical prosthesis, younger than 55 years.^6^

Bleeding occurred in patients of both groups; however, although the use of anticoagulation therapy was more frequent in patients with mechanical prosthesis than bioprosthesis (97.1% vs. 12,1%), bleeding was more frequent in the former group (p=0.0064). No case of fatal bleeding or hemorrhagic stroke was reported. Likewise, in the Veterans Affairs Cooperative Study, 575 patients were randomly assigned to receive either a mechanical valve or a biological one. The risk of bleeding in 11 years was significantly higher in patients with mechanical valves (42% *versus* 26%).^7^

In our cohort, there was no significant difference in the risk of endocarditis between biological and mechanical valve prostheses. The frequency of infections is usually similar between patients with the two types of valve prosthesis during the first postoperative year. In long-term follow-up, the incidence rates of endocarditis in patients with bioprosthesis is comparable to or slightly higher than mechanical prosthesis, although available data are scarce.^8^

The literature has shown a higher incidence of valve prosthesis thrombosis among patients with mechanical than biological prosthesis and highlighted the need for continuous anticoagulation in these patients.^9^This is in accordance with our results, as no statistically significant difference was found in the rate of thrombosis, with an adjusted rate of 5.06% for biological prosthesis and no case recorded for mechanical prosthesis. This may be explained by the fact that our patients were treated in a center specialized in anticoagulation. Chiquette et al.^10^compared treatment with usual medical care and treatment at an anticoagulation center, and reported lower rates of thromboembolic events (minor, major and fatal events).^10^

There was no difference in the risk of ischemic stroke between the two groups (adjusted rate of 14.1% for the biological prosthesis *versu* s 11.5% for mechanical prosthesis at 10 years; p = 0.47). Data in the literature suggested that the risk of thromboembolic complications is usually similar or lower in patients with biological prosthesis compared with patients with mechanical prosthesis and anticoagulation therapy. In an observational study, the cumulative risk of stroke in patients aged between 45 and 54 years undergoing aortic valve replacement was significantly lower (approximately 10% versus 16% at 15 years; HR = 0.64; 95%CI 0.46-0.86; p < 0.05).^6^The increased risk of stroke in our population may be explained by the high prevalence of comorbidities associated, absence of anticoagulation therapy among patients with atrial fibrillation, and lack of prothrombin time control of patients.

In addition, no difference was found in the risk of paravalvular leak between the aortic valves (biological and mechanical). The 10-year rate of paravalvular leak, adjusted for the censored data was 3.38% for the patients with mechanical prosthesis, which is corroborated by the literature, which reports an incidence of 2-10% in patients with aortic valve prostheses. As an example, studies using transesophageal echocardiography after heart valve replacement surgery, the incidence of paravalvular leak varied from 3% to 6%, with a statistical trend for a higher prevalence in patients with mechanical valve prostheses.^11^

In our study, prosthesis-patient mismatch (PPM) was present in 3.76% of patients with bioprosthesis and in no patient with mechanical prosthesis and, for this reason, a simple descriptive analysis was performed. Data in the literature have reported higher incidence of PPM, ranging from 20% to 70%.^12^According to the meta-analysis by the European Heart Journal of 34 studies and a total of 27,186 patients, the presence of PPM was associated with a reduced long term survival (HR = 1.34, 95% CI = 1.18-1.51).^13^IN the comparison between biological and mechanical prostheses, it is probable that bioprosthesis is more prone to PPM, since the effective orifice area of mechanical prostheses is relatively larger due to the area occupied by the suture ring. In patients with a small aortic annulus, the effective orifice area is crucial to improve the hemodynamic performance of the prosthesis, and thereby prevent the occurrence of PPM. In some cases, patients with a small aortic annulus may benefit from a mechanical posthesis.^1^

In the present study, the mean waiting time for surgery was 202 days, with a wide range of distribution, which may be explained by the different indications for surgery and different characteristics of patients.

### Study limitations

One limitation of the present study was its nonrandomized design, which limits the external validity of the results. However, the findings may serve as a basis for further analytical and prospective studies to obtain more consistent conclusions. Other caveats include the fact that this was a single center study, the insufficient sample size for rare events, and loss to follow-up.

In addition, we did not assess reoperation-related mortality, which may have underestimated mortality rates in the bioprosthesis group. Also, prothrombin time data were not evaluated, which makes it difficult to understand the high incidence of ischemic stroke in both groups.

## Conclusion

The probability of overall mortality- and reoperation-free survival in patients with a mean age of 50 years undergoing heart valve replacement surgery in a public tertiary hospital in Sao Paulo State was significantly higher in patients with mechanical valve prosthesis at the expense of a greater durability of this type of prosthesis. No difference was seen in 10-year mortality or in combined adverse events between the groups. A greater need for reoperation was found in patients with bioprosthesis younger than 30 years old. Although no case of fatal bleeding was reported, bleeding was more frequent in patients with mechanical prosthesis than bioprosthesis.
